# High content screen for identifying small-molecule LC3B-localization modulators in a renal cancer cell line

**DOI:** 10.1038/sdata.2018.116

**Published:** 2018-06-26

**Authors:** Likhitha Kolla, David S. Heo, Daniel P. Rosenberg, Sara A. Barlow, Anna A. Maximova, Emily E. Cassio, William J. Buchser

**Affiliations:** 1 College of William & Mary, Department of Biology, Williamsburg, Virginia 23185, USA; 2 Washington University in St Louis, Department of Genetics, St Louis, Missouri 63110, USA

**Keywords:** Autophagy, Cancer screening, High-throughput screening

## Abstract

Forms of selective autophagy have now been recognized to regulate flux in many intracellular processes. Specific pathways and functions have been identified for mitophagy, ERphagy, and other selective autophagies; yet there is no consensus in whether and how autophagy regulates protein maintenance in and around the nucleus. Such processes are of interest for potential degradation of DNA and nuclear envelope proteins in various disease states. The mechanistic details of such nucleus-related autophagic processes remain elusive due to the lack of chemical or genetic regulators to manipulate and follow the process *in vitro*. Here, we describe a high content screen from which we identified small chemical compounds that can modulate the localization of the autophagy marker MAP1LC3B (LC3) in renal carcinoma cells. We also describe a pipeline designed for the execution and analysis of high content screens. The chemical tools discerned from this screen will allow for the deeper exploration of the mechanism, regulation, and molecular targets of nuclear-localized LC3 in perturbed cellular states.

## Background & Summary

Autophagy is a conserved membrane trafficking process that degrades cellular materials to enhance survival^1^. This process was originally seen as a non-selective mechanism activated by general cellular stress such as nutrient deprivation. Recent research has shown, however, that autophagy is additionally responsible for cellular maintenance through the selective degradation of material within specific organelles, including the ER, mitochondria, peroxisomes, and nuclei^2^. Some forms of selective autophagy, such as mitophagy and ER-phagy^2,3^ have been well characterized, while nucleus-related autophagy (nucleophagy) is just becoming recognized. The term nucleophagy may actually represent multiple unique phenomenon associated with the recycling of DNA, nuclear envelope proteins, other nuclear material^4^, micronuclei regulation, or general protein transport dynamics. Autophagy is regularly tracked by LC3 (ATG8, officially MAP1LC3), a microtubule associated protein required for assembly and transport of autophagosomes^5^. LC3B, along with the other autophagy-related isoforms (LC3A, LC3C) are commonly found in and around the nucleus^6^. Here, we perform a high content screen to discover novel chemical tools that specifically modulate nuclear localization of LC3B in a cancer cell line.

In mammalian cells, the process of nucleophagy likely involves the formation of intra- or juxtanuclear autophagosomes containing LC3, nuclear envelope components, and/or DNA which subsequently enter the macroautophagy pathway and fuse with lysosomes^7^ (for review of macroautophagy, see refs 8,9). In disease settings, newly transformed cells upregulate nucleophagy to trigger senescence-induced nuclear envelope remodeling and thereby protect against cancer development^4^. Nucleophagy may also be involved in the nuclear envelope disintegration and recovery during cancer cell migration^4,10^. Similarly, envelopathies, laminopathies, and some neurodegenerative diseases (i.e. Huntington’s) may arise from nucleophagy defects^11–13^.

Other forms of nucleophagy include the engulfment of micronuclei by an autophagosome, or extraction of intranuclear material that may not include the envelope^14^. In the context of amyotrophic lateral sclerosis (ALS), C9orf72 mutations cause the buildup of toxic antisense RNA foci within the nucleus^15^. Nucleophagy is a mechanism for the disposal of these RNA stress granules^16^, and may be important during the onset and progression of disease.

High content screening (HCS) is an automated, microscope-based method used here to discern small compounds that directionally alter the prevalence of cellular phenotypes. We implemented HCS to monitor the effects of small molecules on nuclear morphology and LC3 localization. High content screens are typically coupled with analysis (sometimes termed HCA) to allow for the simultaneous execution and integration of several experiments^17^. Though powerful and widely implemented^18^, HCS is generally difficult and expensive for labs with limited resources.

We adapt several HCS techniques to more standard laboratory equipment and analysis packages then identify a set of small-molecule nuclear LC3 localization modulators. Identified compounds may contribute to the understanding of LC3 nuclear-cytoplasmic transport and nucleophagy in various disease states. On a broader scale, this screen can serve as a template of a widely-accessible microscopy-based pipeline for medium throughput, high content screening in a number of diverse settings.

## Methods

### Cell Culture

786-0 (CRL1932) human cancer cell line from ATCC (Manassas, VA) was cultured in Dulbecco’s Modified Eagle Medium (Thermofisher 11995-065) supplemented with 10% Fetal Bovine Serum (FBS) (Thermofisher 26140079) and 1% PenStrep (Thermofisher 15140-122). Cells were seeded in a 96-well plate at a density of 3,000 cells per well in 100 μL media. Cells were allowed to adhere and divide in a humidified incubator at 37 °C and 5% CO_2_ for 24 h prior to treatment.

### High Content Chemical Screening Assay

1,539 chemical compounds from the NCI DTP diversity set IV were used to identify tools that specifically modulate LC3 localization. Prior to the *in vitro* experiments, the screening compounds were diluted in dimethyl sulfoxide (DMSO) to stock concentrations of 1000 μM and stored in −80 °C. Immediately before experiments, the 1000 μM stocks were further diluted to 80 μM in a 10% DMSO and 90% Phosphate Buffered Saline (PBS) solution, which was used to balance solubility with toxicity of DMSO. 786-0 cells were cultured in a 96 well plate and incubated for 24 h at 37 °C. The border wells of the plate were exposed to a vehicle (DMSO or PBS) and the middle wells to a particular library compound at 8.89 μM. After 4 h of incubation at 37 °C, media was replaced and cells were incubated an additional 18 h before being fixed and stained. Each screening compound was tested in 2-4 independent replicates (different passages of cells from separate days).

### Immunofluorescence staining

#### Expression Analysis

In the screen, LC3B localization was tracked in each cell. To determine the most commonly expressed isoforms, we analysed a publicly available microarray dataset comparing LC3A, LC3B, and LC3C expression across the NCI-60 cell lines [Data Citation 1]. Each cell line had three replicates. The cell lines were ranked (low to high) by log transformed expression values for each isoform of LC3 (Supplementary Table 1). Compared to the other cancer lines, 786-0 cells express a standard amount of both LC3B and LC3C but has less LC3A than other cancer cell lines. In addition to having LC3B expression comparable with most other NCI-60 cancer cells, 786-0 has 4.7 times more LC3B expression than LC3C, making LC3B the dominant isoform.

#### Epitope Analysis

LC3B/MAP1LC3B primary antibody (ThermoFisher, L10382) is more specific to the human LC3B protein than closely related isoforms (MAP1)LC3A and (MAP1)LC3C. The LC3B epitope sequence (PSEKTFKQRRTFEQ) recognized by the antibody was compared against LC3A and LC3C protein sequences below.

LC3A PS**DRP**FKQRR**S**F**AD**

LC3B PS**EKT**FKQRR**T**F**EQ**

LC3C PS**VRP**FKQRK**SLAI**

The bold amino acids indicate unique regions of sequences. While shared regions do exist, there are two distinct linear regions that are unique to each individual isoform.

#### Staining Preparation

786-0 cells were fixed with 3.2% paraformaldehyde (ThermoFisher) in PBS. After rinsing three times with PBS, a solution of 1:1 Bovine Serum Albumin (BSA) to PBS and 0.1% Triton X-100 was added to the plates and incubated at room temperature for one hour to block and permeabilize the cells. LC3 localization was monitored by staining endogenous LC3B with a LC3B/MAP1LC3B primary antibody (ThermoFisher, L10382) at a 1:500 dilution and incubating for 72 h at 4 °C. Subsequently, the primary antibody was rinsed three times with PBS. Goat-Anti-Rabbit IgG Alexa Fluor 546-tagged secondary antibody (ThermoFisher, A-11035) was then added at a 1:1250 dilution and with the nuclear DNA stain with Hoechst (ThermoFisher, H3570) at a 1:5000 dilution. The plates were left at room temperature for one hour, rinsed three times with PBS, and later imaged.

### Microscopy

Experiments were visualized via a Nikon inverted epifluorescent microscope (40X objective) controlled by NIS Elements software in a semi-automated fashion. Prior to imaging, a custom pattern of coordinates was used to move the stage to the center of each well in the 96-well plate. The pattern began at the top left well of the plate and proceeded down the odd columns and up the even columns. After the user manually refined the focus of the Hoechst image, a 2x2 montage was captured around the center point. The Hoechst image was exposed for 400 milliseconds and the LC3 image (with a TRITC filter) for 800 milliseconds. 16-bit, single-channel images were exported from the NIS Elements program as tiff files and subsequently used for image analysis.

### Image Analysis

#### Object Identification

**Figure 1:**
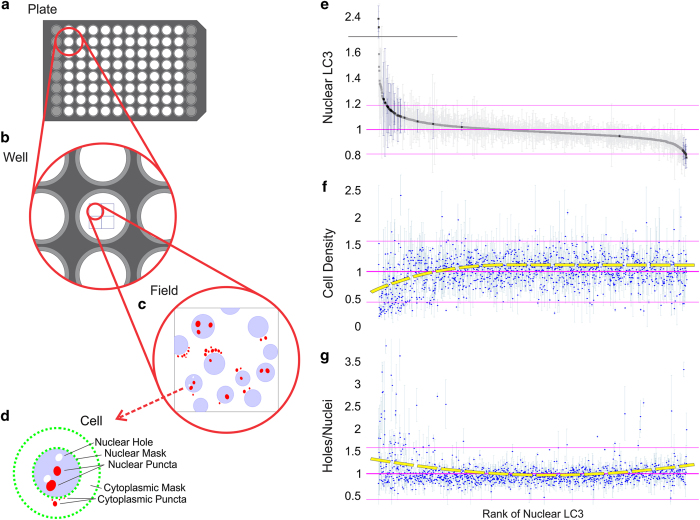
Assay Hierarchy and Tracing and 1,539-Compound Screening Results. (**a**–**d**) Plate>Well>Field>Cell hierarchy. Cells were ‘traced’ and cytometric data was extracted. The green dashed lines indicate the “masks” that define the nucleus (internal ring), and cytosol (external ring). The external ring was created simply by dilating the nuclear mask. The red marks indicate LC3 puncta, which can be localized either in the cytosol or the nucleus. The number, area, and intensity of LC3 within these puncta are measured. Additionally, regions of low DNA (marked by the absence of Hoechst dye) are identified as nuclear holes (a gap in the otherwise blue nucleus). (**e**–**g**) Each data point represents one of the 1,304 chemical compounds that passed quality control. The compounds are ranked by average nuclear LC3 fluorescence (ordered along the x-axis). The x-axes are the same in each graph. Vertical bars represent standard deviation. The three horizontal lines in each plot indicate the global mean of the parameter on the y-axis (middle) +/− two standard deviations. (**e**) Plot of the average nuclear LC3 intensity. Compounds that were selected for the follow-up screen are indicated in dark blue (others are in light gray). (**f**) Ranked compounds plotted against their respective average cell densities. Points that fell below the mean indicate low cell viability and possible toxicity of the chemical. (**g)** Holes per Nuclei (count of nuclear holes). The yellow dashed line in F&G is a curve fit to show the overall relationship between ranked nuclear LC3 intensity and either cell density or nuclear holes.

The stitched 2x2 montage images were run through an ImageJ script, where each image was split into four individual images^19^. The open-source software CellProfiler was used to achieve cell based segmentation by processing individual images containing DNA and LC3 channels^20^. Individual nuclei were traced via Mixture of Gaussians (MoG) thresholding with a cytoplasmic area defined as a set radius of pixels from the nuclear border. The hierarchy of the data is represented in Fig. 1a-d. Intensity, localization, and prevalence of LC3 and DNA were analyzed within the cytoplasm and the nucleus. Additionally, nuclear area and nuclear holes (defined as areas of low DNA content within the nucleus) were measured.

#### Quality Control

Merged RGB jpeg images were exported for visual quality control within *TIBCO Spotfire*. Researchers were blind to all experimental conditions during this phase of quality control. Quality control in Spotfire DecisionSite (TIBCO Spotfire) was achieved by manual examination of each individual image for possible errors in acquisition, tracing of the cells, nuclei, and nuclear holes. In addition to manual vetting of images, we checked for images with low measures of image sharpness (quality), which usually meant the focus was poor. Ultimately, images containing errors in focus, tracing, low viability of 2 cells or less (indicating toxicity), or other artifacts (such as a lint fragment or dye precipitate) were omitted from further analysis.

#### Normalization and Analysis

To adequately compare cells on different plates, data was first normalized so that the global mean of all the wells within a single assay plate for each data parameter was equal to 1. This was done simply by dividing by the global mean of the plate for each parameter separately (implemented with MS Excel). The normalized datasets were then compiled and analyzed. Each set of replicates were compared side by side to access the reliability of the data. *P*-values for significance were extracted from the Spotfire file of the variables of interest. Waterfall plots were constructed by averaging the mean from the independent replicates, which result from the mean of all the cells within that replicate well. Those means were ranked and plotted on the x-axis. In the correlation analysis, replicates were aligned in columns, and MS Excel’s Pearson correlation was used to generate r^2^ coefficients. Linear regression and ANOVA was performed with Spotfire DecisionSite.

### Follow Up Screen

Following the analysis of the initial screen, compounds of interest were selected for follow up dose-response experiments to confirm their validity and determine optimal dosing. Compounds were selected if they significantly increased or decreased the normalized intensity of nuclear LC3 compared to the global average. Additional compounds were included that significantly altered nuclear holes and nuclear area. Compounds of interest were repurchased from the NCI and diluted down to 2000μM in a 10% DMSO and 90% PBS solution. Cells cultured in 96-well plates were exposed to a serial dilution of the compounds ranging from 1.25μM to 20μM (based on the original screening dose of 10μM). Plates were then fixed, stained, and imaged.

### Code availability

Three custom code sets can be accessed at (Figshare) [Data Citation 2]. The first (.xml file) can be used with Nikon NIS Elements AR 3.22 to semi-automatically control the stage and image the 96-well plates. The second (.ijm file) can be used with ImageJ FIJI 1.47a to split the large tiled images down to single images. The final (.cpproj file) is for CellProfiler 2.2.0, to segment the cells within the image. Other software used was *Spotfire* Decision Site 9.1.2 and CorelDraw 15.2.0.

## Data Records

All output data from screens is available on FigShare in ZIP files and spreadsheet format titled “Final_Dataset_Combined” [Data Citation 2].

### High Content, Medium Throughput Screening

1,539 chemical compounds from the NCI DTP Diversity Set IV were used to identify molecules that modulate nuclear LC3 localization. From the 1,304 compounds that passed quality control, further analyses were conducted to determine effects of these on selected cellular parameters, including morphology, nuclear and cytosolic intensity of DNA staining, and nuclear and cytosolic intensity of LC3. Morphological parameters considered include nuclear area, nuclear shape, presence and quantity of LC3 aggregates in nucleus and cytoplasm, and nuclear holes. The majority of compounds had no effect on nuclear LC3 (shaded markers in Fig. 1e). Many compounds that increased nuclear LC3 decreased cell viability (diminution of cell density on the left side of Fig. 1f). Compounds that directionally altered nuclear LC3 slightly increased the abundance of nuclear holes, or areas with low DNA intensity (Fig. 1g), possibly indicating stress or toxicity.

Though 70 compounds significantly affected at least one of the parameters measured (*P*<0.001), 34 were selected (marked in blue in Fig. 1e) for follow-up validation screens due to their significant influence on nuclear and cytoplasmic LC3 intensity as well as abundance of nuclear holes (represented in Fig. 1g). Compounds that exhibited a significant impact on viability were omitted from the validation screen.

### Secondary Screening

**Figure 2:**
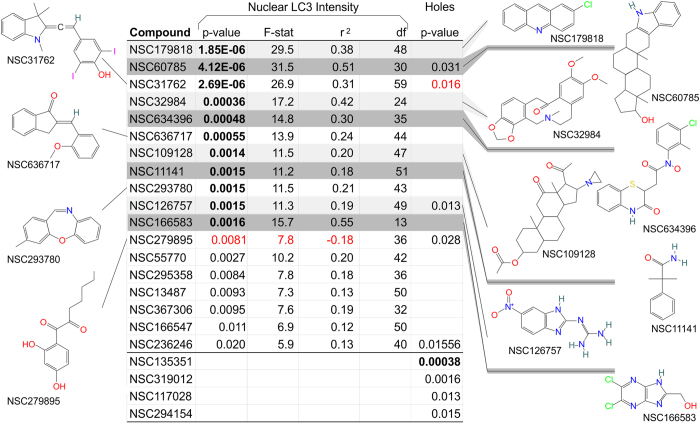
Results from Secondary Screen. The effect of each chemical compound on nuclear LC3 was quantified using a linear regression (dose vs. nuclear LC3 intensity). The *P*-value, F-statistic, r^2^, and degrees of freedom for the regression are shown in the table. Additionally, the *P*-value for a linear regression comparing the dose of the compound to nuclear holes are shown. Bolded *P*-values were still significant after correcting for multiple comparisons by Benjamini Hochberg. Red values represent a negative relationship while black values represent a positive one. Empty cells indicate that a compound did not have significance in this category. The chemical structure of the top twelve hits is displayed along the sides.

Thirty chemical compounds, hereafter referred to as “hits”, were included in a follow-up screen to validate their effects on the nuclear parameters listed above. Cells were subjected to a dose series of each compound. Of these 32 hits, the compounds that significantly altered nuclear LC3 localization are indicated (Fig. 2) and are potentially useful to study LC3-related phenomena like autophagy and nucleophagy.

Seventeen compounds were identified as upregulating nuclear LC3 intensity. Only one compound showed a notable effect on decreasing nuclear LC3 intensity (NSC279895, twelfth row of Fig. 2). After correcting for multiple comparisons by the Benjamin Hochberg test, 11 of the 17 hits (first 11 rows of Fig. 2) maintained their significance in increasing nuclear LC3. Since these experiments started with a high-content screen, we expected many of the initial compounds to be false-positives. We tested the compounds with the dose series to confirm their legitimacy. Eight of the thirty hits significantly increased the nuclear holes with dose (NSC60785, NSC126757, NSC279895, NSC236246, NSC135351, NSC319012, NSC117028 and NSC294154).

Although we are the first to observe the effects of the hits on nuclear LC3 localization, we are not the first to examine these compounds in a cellular assay. PubChem BioAssays revealed some assay findings on hits NSC31762 and NSC279895 among others. NSC31762, the compound inducing the strongest enhancement of nuclear LC3 localization in our screen, has been found active in other cellular assays, notably TRAIL-induced apoptosis [Data Citation 3]. The TRAIL pathway is an innate-immune death pathway known to have cross-talk with autophagy and nuclear import/export^21,22^. NSC279895 [Data Citation 4], the hit compound shown to reduce nuclear LC3 localization, has been characterized as an allosteric enhancer of the Human Thyroid Hormone receptor, implicating nuclear translocation^23^.

## Technical Validation

### Screening Assay Quality

**Figure 3:**
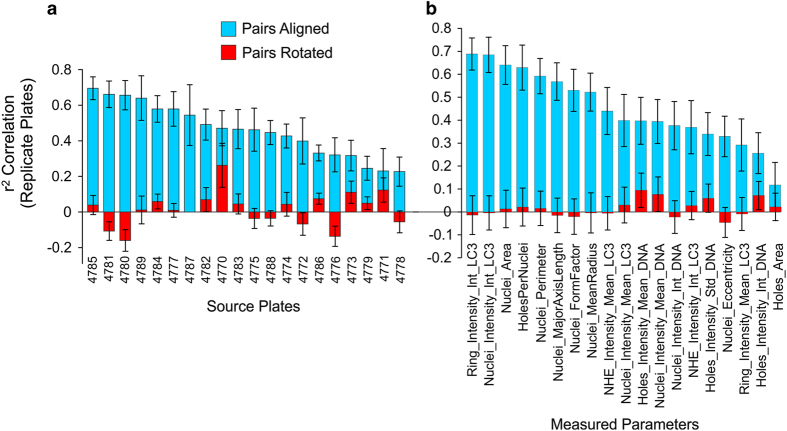
Correlation analysis of plates and parameter. Each pair of plates from the primary screen was analyzed by direct comparison to its replicate plate or by rotating 180 degrees and comparing. **a**. Individual plate IDs are compared well-to-well with their replicate. The displayed value is the average of all the measured parameters (listed in **b**), with standard deviation. Blue bars are comparison of like treatments (for example B3 to B3, F5 to F5, making for a high correlation), while red bars are comparison of the treatment to its 180 degree rotated partner (B3 to G10, F5 to C8). X-axis is ordered so that the plates with the best correlations are on the left. **b**. Correlations for measured parameters (ranked best to worst), averaged across all plates.

A technical pre-validation was run before analyzing the primary screen data. We performed a correlation analysis between replicate plates^24^. Each treatment was tested for correlation among sets of replicate plates; with a high r^2^ indicating the reliability of the drug affecting a particular cellular parameter(s). Correlation values closer to zero signify noisy data and a lack of consistency between compound effects for replicates of the same assay plate. Among the source plates used (Fig. 3a), all but two had adequate correlation. Among the various parameters measured (Fig. 3b), LC3 intensity measurements had extremely good repeatability, with other measurements being adequate. Covariance analysis provided insight into the cellular variables that were affected the most by the compound. Cytoplasmic LC3, nuclear LC3, nuclear area and holes per nuclei were four of the most robust parameters across all plates.

It is common for multi-well plates to suffer from variations in phenotype across the plate (referred to as *plate effects*), due to differences in temperature and evaporation, especially comparing the edge wells to the more central wells^25^. Strong plate effects result in a correlation that is artificially high; sections of the plate are compared rather than the treatments. To account for this, a second analysis was done after ‘rotating’ one of the two replicate plates 180 degrees, then overlaying it on its complementary replicate plate (Fig. 3a,b, red bars). As a result, the compounds were no longer aligned and resulting high r^2^ values would indicate strong plate effects. From this analysis, we find that most effects observed were due to the treatment (chemical compound), and not plate effects.

Another technical validation is useful to consider when running compound screens with fluorescent readouts. Some of the compounds may possess chemical structures that naturally fluorescence at various wavelengths due to conjugated aromatic rings. It may be useful to assess the chemicals’ optical properties before using them *in vitro* to manipulate cellular phenotypes, allowing for the differentiation of probe intensities from the underlying auto-fluorescence of the chemicals. In this screen, we checked for compound fluorescence empirically, but that may be avoided by using the third-party analysis program like Hyperchem to predict the fluorescence spectra of the compounds^26^.

## Usage Notes

The high content, medium throughput screening method described here is a useful alternative to the established, yet expensive and technically complicated, high throughput process. We utilized free open-source analysis software packages *ImageJ* and *CellProfiler* to analyze functional cellular parameters. We used our screening pipeline to discern nuclear LC3 localization modulators, however, the applications of this method extend beyond our results. The pipeline can be implemented for other chemical libraries to assess the directional influence of a wide array of substances on diverse cellular parameters.

Our screen identifies chemicals that may modulate nuclear-associated types of autophagy. Most forms of autophagy require the formation of an autophagosome and later fusion of the autophagosome with a lysosome for degradation of intra-vesicular material. The screening assay here measures nuclear LC3 localization, but it does not test for the autophagic flux into the lysosome.

The novel tools discerned from this screen could be used to understand how nuclear stress and potentially nucleophagy may alter the cellular phenotype of cells undergoing a variety of stress conditions in systems beyond cancer. One area of strong interest may be the C9ORF72 mutation implicated in ALS and FTD, which is known to induce stress-causing RNA foci within the nucleus that may require clearance by nucleophagy. We hope these chemicals will allow those examining nuclear stress, transport, and degradation to answer questions about the substrates, receptors, and interacting pathways and their function involved in these complex processes.

## Additional information

**How to cite this article**: Kolla, L. *et al*. High content screen for identifying small-molecule LC3B-localization modulators in a renal cancer cell line. *Sci. Data* 5:180116 doi: 10.1038/sdata.2018.1116 (2018).

**Publisher’s note**: Springer Nature remains neutral with regard to jurisdictional claims in published maps and institutional affiliations.

## Supplementary Material



Supplementary Information
