# Supplementary material for: High content screen for identifying small-molecule LC3B-localization modulators in a renal cancer cell line
Source: Sci Data. 2018 Jun 26;5:180116. doi: 10.1038/sdata.2018.116 (PMC6018519; doi:10.1038/sdata.2018.116)
Supplement: Supplementary Information [file sdata2018116-s2.docx]

**High content screen for identifying small-molecule LC3B-localization modulators in a renal cancer cell line**

## Supplement

**Supplemental Table 1**

|  | Min | Max | Mean | StdDev |
| --- | --- | --- | --- | --- |
| LC3A | 19% | 25% | **23%** | 3% |
| LC3B | 37% | 46% | **43%** | 4% |
| LC3C | 42% | 78% | **55%** | 16% |

LC3 mRNA expression was measured with NCI-60 public microarray data [Data Citation 1]. The values in each cell of the table represent the percentile value of 786-0 cells compared to the 60 other types of cells in the NCI-60 set. The first well can be read as follows: 786-0 are at a minimum of 19 percentile for LC3A expression, so they are part of the lowest quartile. LC3A expression in 786-0 renal carcinoma cells, ranged between 19-25 percentile. LC3B expression for the 786-0 cell line was 37-46 percentile and LC3C expression was in the 42-78 percentile range. LC3B has a near-median percentile in 786-0 and its ranking among the three replicates shows low variability.
